# Prevalence of stroke survival in rural communities living in northern Peru

**DOI:** 10.1371/journal.pone.0254440

**Published:** 2021-07-29

**Authors:** Luz M. Moyano, Silvia M. Montano, Percy Vilchez Barreto, Narcisa Reto, Luis Larrauri, Nicanor Mori, Mario Cornejo-Olivas, Erik Guevara-Silva, Fernando Urizar, Enrique Najar, Ricardo Gamboa, Cintya Azabache, Raquel Herrer Ticse, Lucia Bolivar-Herrada, Alex Doud, Peggy Martinez, J. Jaime Miranda, Joseph R. Zunt, Hector H. García, for the Cysticercosis Working Group for Peru

**Affiliations:** 1 Cysticercosis Elimination Program, Center for Global Health, Universidad Peruana Cayetano Heredia, Tumbes, Perú; 2 Sección de Epidemiologia, Instituto de Medicina Tropical Daniel Alcides carrión Universidad Nacional Mayor de San Marcos, Lima, Perú; 3 Centro Basico de Investigación en Demencia y enfermedades desmielinizantes, Instituto Nacional de Ciencias Neurológicas, Lima, Perú; 4 Departamento de Medicina, Hospital Nacional Daniel Alcides Carriòn, Lima, Perú; 5 Neurogenetics Research center (MC), Instituto Nacional de Ciencias Neurológicas, Lima, Perú; 6 Departamento de Medicina, Hospital Nacional Cayetano Heredia, Lima, Perú; 7 Departments of Neurology, Global Health, Medicine and Epidemiology, University of Washington, Seattle, WA, United State of America; 8 Departamento de Pediatría, Instituto Nacional del Niño, San Borja, Lima, Perú; 9 CRONICAS Center of Excellence in Chronic Diseases, Universidad Peruana Cayetano Heredia, Lima, Perú; Nicolaus Copernicus University in Torun: Uniwersytet Mikolaja Kopernika w Toruniu, POLAND

## Abstract

**Background and purpose:**

Stroke is the leading cause of neurological impairment in the South American Andean region. However, the epidemiology of stroke in the region has been poorly characterized.

**Methods:**

We conducted a staged three-phase population-based study applying a validated eight-question neurological survey in 80 rural villages in Tumbes, northern Peru, then confirmed presence or absence of stroke through a neurologist’s examination to estimate the prevalence of stroke.

**Results:**

Our survey covered 90% of the population (22,278/24,854 individuals, mean age 30±21.28, 48.45% females), and prevalence of stroke was 7.05/1,000 inhabitants. After direct standardization to WHO’s world standard population, adjusted prevalence of stroke was 6.94/1,000 inhabitants. Participants aged ≥85 years had higher stroke prevalence (>50/1000 inhabitants) compared to other stratified ages, and some unusual cases of stroke were found among individuals aged 25–34 years. The lowest age reported for a first stroke event was 16.8 years. High blood pressure (aPR 4.2 [2.7–6.4], *p>0*.*001*), and sedentary lifestyle (aPR 1.6 [1.0–2.6], *p = 0*.*045*) were more prevalent in people with stroke.

**Conclusions:**

The age-standardized prevalence of stroke in this rural coastal Peruvian population was slightly higher than previously reported in studies from surrounding rural South American settings, but lower than in rural African and Asian regions. The death rate from stroke was much higher than in industrialized and middle-income countries.

## Introduction

Stroke is the leading cause of neurological impairment in South America’s Andean region, which includes Bolivia, Colombia, Ecuador, Peru, and Venezuela [1]. Despite this significant cerebrovascular disease burden, there is little information regarding stroke rates, mortality, or associated risk factors in rural populations [2, 3].

The World Health Organization Monitoring Trends and Determinants in Cardiovascular Disease (MONICA) project does not include stroke registry information for any country in the Andean region [4]. In 1988, a population-based study conducted in Cuzco, Peru, showed an age-adjusted prevalence of 5.74 per 1,000, [5] similar to Colombia (5.6/1,000), [3] but slightly higher than Bolivia (3.22/1,000) [6]. Compared with other regions worldwide, Latin America has higher proportions of patients with hemorrhagic stroke [7], small vessel disease [2, 7, 8] and intracranial atherosclerotic lesions [9]. Additionally, the Andean sub-region possesses unique infectious risk factors, including Chagas’ disease, neurocysticercosis, malaria, leptospirosis and viral hemorrhagic fevers, and non-infectious factors such as high altitude hypoxia and snake bites [2–5, 10, 11].

Taking advantage of a prior population-based epidemiologic survey from a cysticercosis elimination program in Tumbes, Peru, we applied a validated eight-question neurological survey to estimate stroke symptom prevalence, confirm presence of stroke through a standardized neurologic examination, and evaluate stroke-associated risk factors in this region.

## Materials and methods

### Study population

**Fig 1:**
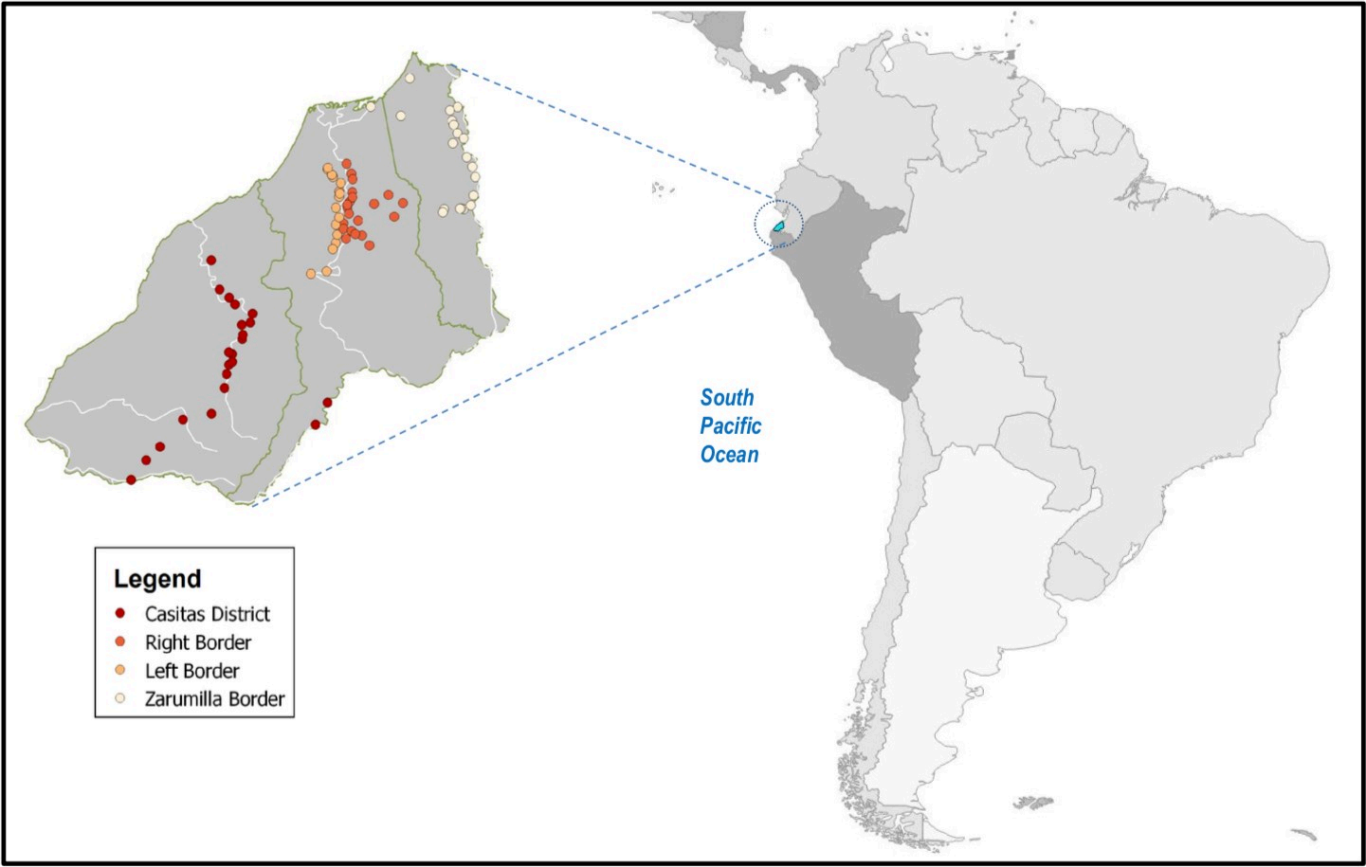
Map of 80 rural communities in Tumbes involved in the study.

We cross-sectionally surveyed 24,854 individuals from 80 communities from the cysticercosis elimination program, living near sea-level along Peru’s Northern coast (Fig 1). The study area, covering 4,669.2 km2, contains a mostly Mestizo population. Life Expectance in Peru from 2015–2020 was 76,26 years old. Most villages have electricity but lack sewage facilities or running water. The area has 28 basic-level health centers; each staffed by a recently-graduated general practitioner (GP) performing a one-year rural service; one nurse and one health worker. Activities were performed in three phases.

### Phase I–Stroke survey

A baseline census was performed to obtain household-level information. After obtaining informed consent (IC), non-medical field workers (trained by a team of neurologists) administered an eight-question face-to-face survey to identify stroke symptoms in all individuals older than 15 years. Illiterate individuals were included through reading aloud the IC and survey to ensure understanding. This WHO stroke detection questionnaire was translated into Spanish, modified, and validated by Del Brutto *et al*. in the Atahualpa Project. [12] Most older individuals were living in a relative’s home.

### Phase II–General practitioner evaluation

Participants reporting symptoms of stroke by responding yes to question 1 or 2, or a combination of two positive responses between questions 3 to 8, were evaluated in a local health center by a GP trained by neurologists to recognize stroke and other conditions mimicking stroke. Care was taken to include the local dialect to describe stroke, such as *“derrame”*. The evaluation included anthropometric measures (weight, height, body mass index (BMI) calculated as weight (kg) divided by squared height (m2) [13], abdominal circumference, and brachial circumference), as well as questions regarding history of high blood pressure, diabetes, dyslipidemia, cardiac disease, smoking, alcohol or illicit drug use, and amount of exercise. All participants classified as *“suspected stroke”* were included in Phase III (neurological evaluation) to confirm diagnosis of stroke.

### Phase III—Neurological examination, blood testing and brain computerized tomography (CT) scan

A team of board-certified neurologists evaluated suspected stroke cases for case-confirmation and to rule out non-stroke events. Stroke was defined according to WHO criteria, as “*rapid development of clinical signs of focal (or global) disturbance of cerebral functions*, *lasting >24 hours*, *with no apparent cause other than vascular*” [6, 12, 14–16]. Non-contrast brain CT scans were offered to all participants with a suspected diagnosis of stroke and performed using a helicoid CT scan (Siemens AG, Germany) in the Center for Global Health facility. Reproductive-aged women had a urine pregnancy test performed prior to imaging, and pregnant women did not undergo brain CT.

Venous blood samples (8ml) were collected from stroke cases to assess: fasting blood glucose (FBG), glycosylated hemoglobin (HbA1c), serum lipid profiles including total cholesterol (TC), triglyceride (TG), The total cholesterol/HDL cholesterol ratio (American Heart Association [AHA] [17] target below 5 in males, and below 4.4 in females), the LDL cholesterol/HDL cholesterol ratios (AHA target below 3.5 in males, and below 3.2 in females) [17] and hematocrit.

Hypertension was defined as mean systolic blood pressure (SBP) ≥140mmHg or mean diastolic blood pressure (DBP) ≥90mmHg [13], diabetes mellitus (DM) was diagnosed as FBG ≥126 mg/dl or HbA1c ≥6.5% [18] and/or self-reported diagnosis during the GP evaluation. Heart disease history was self-reported during the GP evaluation. Participants were asked whether they regularly used tobacco (current smoking defined as ≥1 cigarette/day) [13], consumed alcohol (any alcoholic drink in the last week), sedentary lifestyle (office work, driving as a chauffeur, and sitting while watching television or low physical activities) [19].

### Ethical considerations

The study protocol and consent forms were approved by the institutional review boards of Universidad Peruana Cayetano Heredia, the University of Washington and the Regional Directorate of the Ministry of Health (DIRESA) in Tumbes.

### Statistical analysis

Prevalence of stroke was defined as number of persons with confirmed stroke divided by number of baseline survey respondents. Age-adjusted stroke prevalence was obtained by dividing the number of people with stroke in each age bracket by the number of individuals in the same age bracket as stratified in the World (WHO 2000–2025) standard population. Incidence was defined as number of persons who developed stroke in the year preceding the prevalence day (April 5, 2011), divided by total number of study participants. The world (WHO 2000–2025) standard population census was the reference population for prevalence age adjustments. Confidence intervals were estimated using exact binomial method. Prevalence ratios (PR) and adjusted PR (aPR) were estimated using Poisson family general linear models with logit link. All reported probability (p) values were two-sided with significance level set at 0.05. Variables significant at the level of *p*<0.25 were retained in the multivariable models from which adjusted odds ratios were estimated. All-cause mortality was defined as total number of deaths during the study period (between phase I and III; April 2011-May 2012), divided by participant population. We used Stata version 14.2 for statistical analysis (Stata Corp., College Station, TX, USA).

## Results

From a population of 34,825 people across 80 rural communities, 24,854 individuals older than 15 years were invited to participate; 22,278 (89.63%) individuals provided informed consent and completed the eight-question stroke survey. The participants most frequently lived in hand-constructed mud and cane houses, had public potable water service that was functional for a few hours each day, treated water with chlorine pills, and defecated in holes/silos; although a sizable proportion (24.2%) reported open field defecation.

### Comparison between participants and non-participants

Compared to study participants, a larger proportion of non-participants were male (1,891/2,577 [73.4%] vs 11,074/22,278 [49.7%]; *p<0*.*0001*); slightly younger (37.1 ± 17.2 vs 39.9 ± 17.9; *p<*0. 0001); and lived in multi-family households (564/2,577 [21.9%] versus 3,937/22,278 [17.7%]; *p<0*.*0001*). There were no differences in urbanicity, housing material, water sources, drinking water, bathroom, and electricity between groups.

### Comparison between positive and negative survey respondents

The proportion of positive respondents to the survey was 7.1% (1,586/22,278). Positive respondents were more frequently female (920/1,586 [58.0%] vs 10,284/20,692 [49.7%]; *p<0*,*0001*), older (mean age 47.9 ± 19.1 years vs. 39.3 ± 17.6 years; *p<0*,*0001*), and used latrines in a slightly higher proportion (1,270/1,586 [80.1%] vs 16,038/20,692 [77.5%], *p = 0*,*019*) than negative respondents. The groups were similar in relation to housing material, household, water sources, drinking water and electricity (Table 1).

**Table 1:** Demographic characteristics of respondent’s vs non-respondents and between negative and positive individuals surveyed for stroke.

Demographic characteristics and categories	Non-respondents (n = 2577)		Respondents (n = 22,278)		*p-value*	Negative survey (n = 20,692)		Positive survey (n = 1,586)		*p-value*
	Number	%	Number	%		Number	%	Number	%	
**Sex**					** **					** **
Female	686	26.6	11,204	50.3	**<0,0001**	10,284	49.7	920	58.0	**<0,0001**
Male	1,891	73.4	11,074	49.7	** **	10,408	50.3	666	42.0	** **
**Age** ^**a**^					** **					** **
Mean + sd (IC) ^b^	37,8 ± 17,2	37,1–38,5	39,9 ± 17,9	39,7–40,2	**<0,0001**	39,3 ± 17,6	39,1–39,6	47,9 ± 19,1	46,9–48,8	**<0,0001**
**Urbanity**					** **					** **
Marginal Urban	235	9.1	2,034	9.1	**= 0,985**	1,907	9.2	127	8.0	**= 0,107**
Rural	2,342	90.9	20,244	90.9	** **	18,785	90.8	1,459	92.0	** **
**Material of Housing**					** **					** **
Brick and others	435	16.9	4,066	18.2	**= 0,087**	3,803	18.4	263	16.6	**= 0,074**
mud and cane	2,142	83.1	18,212	81.8	** **	16,889	81.6	1,323	83.4	** **
**Housing condition**					** **					** **
Own home	2,488	96.5	21,191	95.1	**= 0,005**	19,666	95.0	1,525	96.2	**= 0,137**
Rent	46	1.8	537	2.4	** **	506	2.5	31	1.9	** **
Other	43	1.7	550	2.5	** **	520	2.5	30	1.9	** **
**Household**					** **					** **
Singlefamily	2,013	78.1	18,341	82.3	**<0,0001**	17,052	82.4	1,289	81.3	**= 0,253**
Multifamily	564	21.9	3,937	17.7	** **	3,640	17.6	297	18.7	** **
**Source of water**					** **					** **
Public service	2,131	82.7	18,752	84.2	**= 0,052**	17,438	84.3	1,314	82.8	**= 0,134**
Non public service	446	17.3	3,526	15.8	** **	3,254	15.7	272	17.2	** **
**Drinking Water**					** **					** **
Artesanal Treatment	2,066	80.2	17,672	79.3	**= 0,315**	16,425	79.4	1,247	78.4	**= 0.046**
Untreated	511	19.8	4,606	20.7	** **	4,267	20.6	339	21.6	** **
**Bathroom**					** **					** **
Bath	472	18.3	3,759	16.9	**= 0,079**	3,467	16.8	292	18.4	**= 0,031**
Latrine	1,568	60.9	13,549	60.8	** **	12,571	60.7	978	61.7	** **
Did not have	537	20.8	4,970	22.3	** **	4,654	22.5	316	19.9	** **
**Electricity**					** **					** **
On electric grid	2,388	92.7	20,643	92.7	**= 0,993**	19,193	92.8	1,450	91.4	**= 0,050**
no electricity	189	7.3	1,635	7.3	** **	1,499	7.2	136	8.6	** **

^a^ mean and standard deviation

^b^ p- value were calculated using Student’s t test

### Prevalence of stroke survival

**Fig 2:**
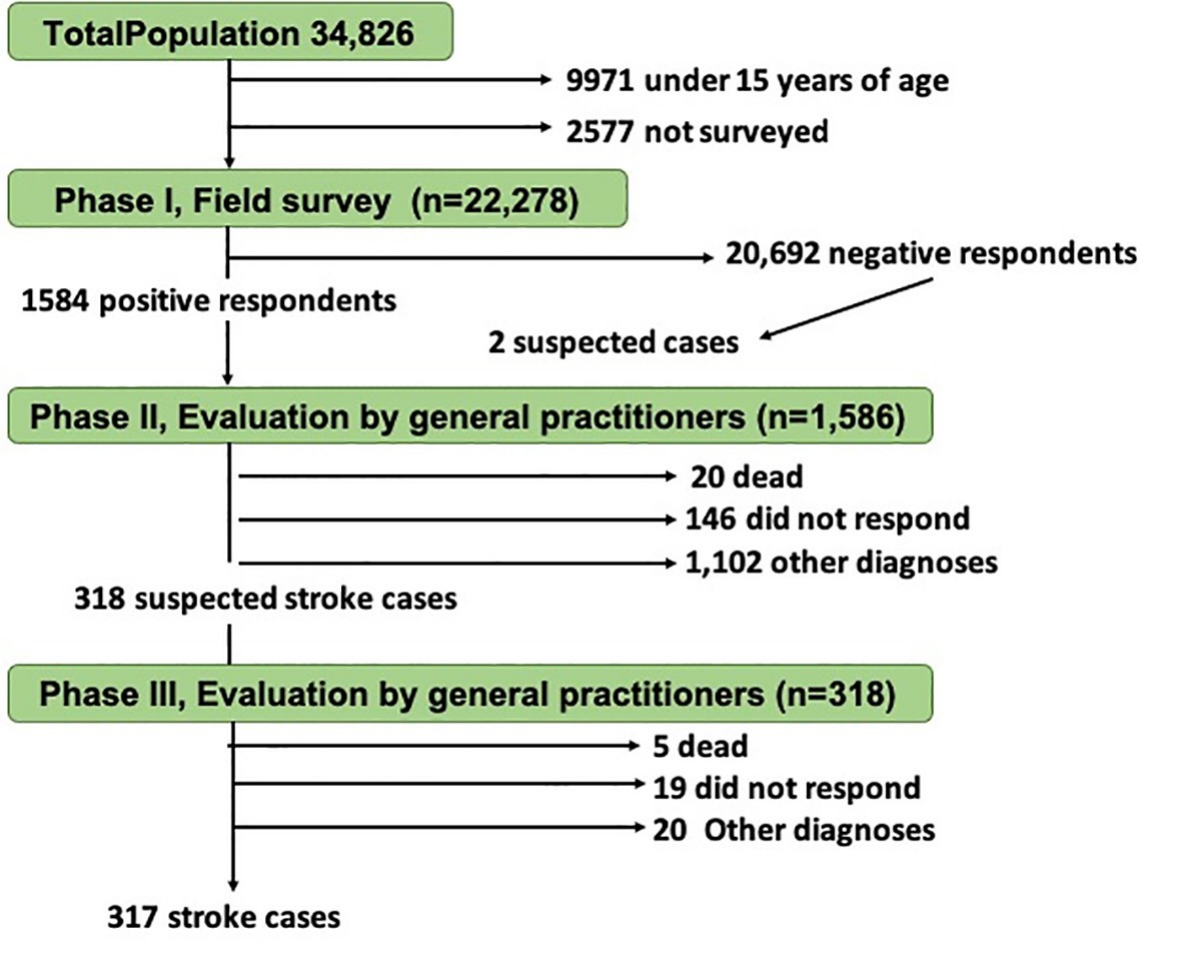
Study flowchart.

A total of 1,420 individuals were evaluated by a study physician (including two negative survey respondents self-referred by study physicians due to symptoms compatible with stroke). We classified these 318/1,420 (2.2%) as “*suspected of stroke*”, and 1,102 had a diagnosis other than stroke (Fig 2).

**Fig 3:**
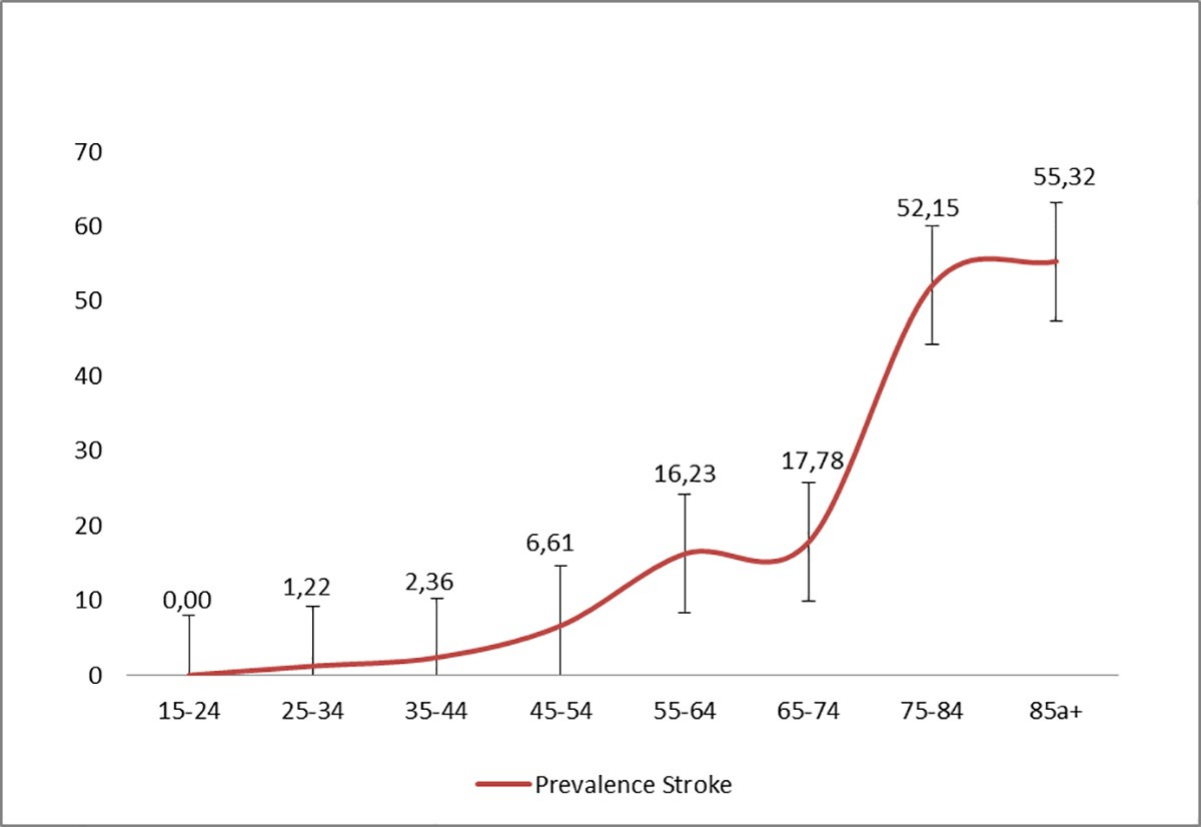
Prevalence of stroke and confidence interval stratified by age in 80 rural communities.

Of the 318 participants with suspected stroke, 24 (7.5%) declined the neurologist evaluation, 137 had a diagnosis other than stroke, and 157 individuals were confirmed as having had a stroke event. Stroke survival prevalence was 7.05/1,000 (157/22,278) inhabitants. After direct standardization to WHO’s world standard population, the adjusted prevalence of stroke was 6.94/1,000 inhabitants. Participants aged 85 years and older had much higher prevalence (>50/1,000 inhabitants) compared with other stratified ages, and some unusual cases were found in individuals aged 25–34 years (Fig 3). Crude prevalence was similar for men and women (81/11,204, 7.23 per 1,000 men vs 76/11,074, 6.86 per 1,000 women; *p = 0*.*636*) (Table 2).

**Table 2:** Age-adjusted and sex specific prevalence of 80 rural communities in the northern coast of Peru.

Age—Group	Population			Men			Women			Prevalence / 1000 habitants	Population			Prevalence adjusted / 1000 ^a^			
	Number	%	People With Stroke	Number	People With Stroke-Men	Prevalence / 1000	Number	People With Stroke-Women	Prevalence / 1000		Number	% INEI 2017	% WHO	WHO	Tumbes	Men	Female
15–24	5121	22,99	0	2666	0	0,00	2455	0	0,00	**0,00**	35799	22,52	22,50	**0,00**	**0,00**	**0,00**	**0,00**
25–34	4905	22,02	6	2631	2	0,76	2274	4	1,76	**1,22**	35096	22,08	21,35	**1,19**	**1,23**	**0,75**	**2,14**
35–44	4245	19,05	10	2137	3	1,40	2108	7	3,32	**2,36**	31253	19,66	18,52	**2,29**	**2,43**	**1,43**	**3,44**
45–54	3327	14,93	22	1619	9	5,56	1708	13	7,61	**6,61**	24698	15,54	15,38	**6,81**	**6,88**	**5,41**	**6,47**
55–64	2157	9,68	35	1043	18	17,26	1114	17	15,26	**16,23**	17550	11,04	11,15	**18,68**	**18,50**	**13,85**	**10,51**
65–74	1406	6,31	25	655	13	19,85	751	12	15,98	**17,78**	8732	5,49	6,97	**19,64**	**15,48**	**14,34**	**9,57**
75–84	882	3,96	46	349	29	83,09	533	17	31,89	**52,15**	4284	2,70	3,28	**43,15**	**35,51**	**61,18**	**13,68**
85a+	235	1,05	13	104	7	67,31	131	6	45,80	**55,32**	1535	0,97	0,85	**44,54**	**50,65**	**51,07**	**29,48**
**Total**	**22278**	**100**	**157**	**11204**	**81**	**7,23**	**11074**	**76**	**6,86**	**7,05**	**158947**	**100,00**	**100,00**	**6,94**	**6,47**	**5,74**	**4,80**

^a^ Information from censuses of 2017 (National Institute of Statistic and informatics (INEI)

The youngest age reported for first-stroke event was 16.8 years; mean age at first event was 58.9 ± 16.5 years (mean ± SD), median 63 years, and interquartile range [IQR] 47.5–78.5 years. In age groups, first-stroke events were between 15 and 25 in 5 (3.9%), between 26 and 35 years in 10 (6.5%), between 36 and 45 years in 18 (11.6%), between 46 and 55 years in 30 (19.4%), between 56 and 65 years in 28 (18.1%), between 66 and 75 years in 39 (25.2%), between 76 and 85 years in 21 (13.5%), and after 85 years in 3 (1.9%). Two people with stroke (PWS) did not remember the date of their stroke event. Fifteen PWS (9.8%) had a history of second stroke event and three (2.0%) had a history of third event.

### All-cause mortality

Ninety-five study participants died between phases I and III (426.42 /100,000 population per year). Of these 95 deaths, 25 were people classified as “*suspected stroke*” (25/1586, [1.6%]); 4 in positive survey respondents, but not suspected of stroke (4/1266, [0.3%]), and 66 in negative survey respondents (66/20,692, [0.3%]). The corresponding death rates were 1,576/100,000 population per year in people suspected of stroke and 316/100,000 population per year in the other two groups (*p< 0*.*0001*).

### Other neurological conditions

Other relevant neurological diagnoses in non-stroke cases included epilepsy in 84 participants, Bell’s palsy in 152 participants, transient ischemic attack (TIA) in 37 participants, 2 cases with sequelae of Guillain-Barre, and 2 cases of amyotrophic lateral sclerosis.

### Retrospective stroke incidence

Eighteen stroke events occurred in the year before the survey, for a crude annual incidence rate of 80.80 per 100,000 person/years. The incidence rate adjusted to 2007 census age distribution [20] was 129.72 per 100,000 person/years.

### Clinical sub-types of stroke and brain CT findings

From the 157 PWS, clinical history of ischemic stroke was found in 139 (88.5%; 66 females and 73 males, mean age 66, median age 68, range of time-lapse of event between 0–49 years, average 7.10 years) and hemorrhagic stroke in 21 (11.6%; 12 females and 9 males, mean age 63, median age 61, range of time-lapse of event between 1–25 years, average 9.33 years). Three (1.9%) individuals with ischemic stroke later developed hemorrhagic stroke.

Non-contrast brain CT scan [21] was obtained in 132 PWS, 116 (87%) of whom had a ischemic stroke diagnosis and 16 (23%) hemorrhagic stroke. Encephalomalacia was found in 59 (44.4%; [50/59 individuals with ischemic stroke]), atrophy with associated leukoencephalopathy [22] in 28 (21.1%; [24/28 individuals with ischemic stroke]) and normal findings in 18 (13.5%; all with ischemic stroke).

### Morbidity and characteristics of PWS

Differences in self-reported cardiovascular information between PWS (n = 157) and people without stroke (PWOS, n = 1,102) included non-modifiable factors such as gender and age, with PWS more frequently male (81/157[52.3%] vs 424/1,102 [38.5%], *p = 0*.*002*), and older (65.5±15.7 vs 45.6±18.1 years, *p = 0*.*0001*). Modifiable factors such as high blood pressure, diabetes, dyslipidemia, history of heart disease, tobacco use, and sedentary lifestyle differed significantly between PWS and PWOS (Table 3): in an age- and sex-adjusted model, both high blood pressure (aPR 4.2 [2.7–6.4], *p>0*.*001*), and sedentary lifestyle (aPR 1.6 [1.0–2.6], *p = 0*.*045*), were significantly more prevalent in PWS (Table 3).

**Table 3:** Self reported information about cardiovascular health between stroke cases and non-stroke cases.

Demographic characteristics and categories	Non-stroke (n = 1102)		Stroke (n = 157)		Crude model		Adjusted model ^b^	
					Prevalence Ratio	*p-value* ^a^	Adjusted Prevalence Ratio	*p-value* ^a^
Sex								
Female	678	61.52	76	48.41	Ref.	Ref.	Ref.	Ref
Male	424	38.48	81	51.59	1,591 (1,188–2,131)	= 0,002	1,429 (0,972–2,101)	= 0,070
Age ^b^	** **	** **	** **	** **				
Mean + standard deviation	45,0 ± 18,2		65,5 ± 15,7		1,046 (1,039–1,053)	<0,0001	1,028 (1,017–1,039)	<0,0001
**Self-reporting information**								
Hypertension								
Absent	867	78.7	38	24.2	Ref.	Ref.	Ref.	Ref
Present	235	21.3	119	75.8	8,006 (5,676–11,292)	<0,0001	4,221 (2,775–6,421)	<0,0001
Body mass index		** **	** **	** **	** **	** **	** **	** **
Normal	433	39.3	53	33.8	Ref.	Ref.	Ref.	Ref
Underweight	17	1.5	5	3.2	2,084 (0,926–4,691)	= 0,076	1,512 (0,596–3,845)	= 0,383
Overweight	434	39.4	69	43.9	1,258 (0,899–1,759)	= 0,180	1,161 (0,805–1,674)	= 0,424
Obese	218	19.8	30	19.1	1,109 (0,728–1,690)	= 0,629	1,115 (0,691–1,801)	= 0,655
Diabetes Mellitus	** **	** **	** **	** **	** **	** **	** **	** **
Absent	1,035	93.9	138	87.9	Ref.	Ref.		
Present	67	6.1	19	12.1	1,878 (1,226–2,877)	= 0,004	1,126 (0,679–1,866)	= 0,646
Dislipidemia		** **	** **	** **	** **	** **	** **	** **
Absent	907	82.3	102	65.0	Ref.	Ref.		
Present	195	17.7	55	35.0	2,176 (1,617–2,929)	<0,0001	1,210 (0,844–1,735)	= 0,300
Heart Disease		** **	** **	** **	** **	** **		
Absent	1,080	98.0	148	94.3	Ref.	Ref.		
Present	22	2.0	9	5.7	2,409 (1,361–4,263)	= 0,003	1,096 (0,550–2,185)	= 0,795
Tobacco use		** **	** **	** **	** **	** **	** **	** **
None	710	64.4	82	52.2	Ref.	Ref.		
Yes	392	35.6	75	47.8	1,551 (1,159–2,076)	= 0,003	1,221 (0,825–1,807)	= 0,318
Alcohol consumption	** **	** **	** **	** **	** **	** **	** **	** **
None	244	22.1	34	21.7	Ref.	Ref.		
Yes	858	77.9	123	78.3	1,025 (0,718–1,463)	= 0,891	0,836 (0,544–1,285)	= 0,414
Consumption of drugs		** **	** **	** **	** **	** **	** **	** **
None	1,082	98.2	152	96.8	Ref.	Ref.		
Yes	20	1.8	5	3.2	1,624 (0,731–3,606)	= 0,234	1,055 (0,425–2,616)	= 0,908
Regular exercise		** **	** **	** **	** **	** **	** **	** **
Yes	304	28.3	19	12.1	Ref.	Ref.		
None	798	71.7	138	87.9	2,506 (1,578–3,981)	<0,0001	1,647 (1,010–2,685)	= 0,045

^a^ p- value were calculated using glm link(log) fam(Poisson)

^b^ adjusted model by sex and age

### Laboratory results in PWS

Blood samples were obtained from 122 PWS to measure cholesterol, VLDL, HDL, triglycerides, glycosylated hemoglobin (HbA1c), fasting glucose and hematocrit. Of these, HbA1c > 6% was detected in 28 (22.9%), intermediate cholesterol levels (between 200–239 mg/dl) were found in 40 (32.8%), and high cholesterol levels (> 240mg/dl) were found in 35 (28.7%). Borderline high triglycerides (150–199 mg/dl) were present in 29 (23.8%), a high level (200–499 mg/dl) in 15 (12.3%) and very high level (>500 mg/dl) in 2 (1.6%). Total cholesterol/HDL cholesterol ratios were outside of normal ranges in 25 males (20.5%) and 32 females (26.2%); LDL cholesterol/HDL cholesterol ratios were outside of normal ranges in 47 males (38.5%) and 35 females (28.7%). Fasting glucose levels were > 126 mg/dl in 12/122 (9.4%), and low hematocrit levels (below 38%) were present in 20/122 individuals (16.4%).

## Discussion

Although stroke is preventable, and international efforts, such as MONICA [23] and INTERSTROKE [24], were performed to reduce stroke risk factors, in the past 3 decades our study is only the second wide-scale neuroepidemiological study of stroke in a rural setting (Tumbes, Peru) and the first involving >20,000 inhabitants [3]. In this population-based study, the crude prevalence of stroke survival was 7.05/1,000 in rural Peru—higher than in other community-based studies in South America (Bolivia 1.74/1,000 [6], Ecuador 3.6/1,000 [3], Colombia 4.7/1,000 [25]), Central America (Honduras 3.6/1,000 [26]) and África (Southern Nigeria 1.63/1,000 [27]); slightly higher than in Cusco (Peru, Highland) 6.2/1,000 [5] and lower than in Asia (China 66.90/1,000 [13] and 15.96/1000 [28]) and other African studies (Nigeria, Delta Region 13.31/1,000 [29] and 8.51/1,000 [30]). We also detected a worrisome high prevalence of stroke in people younger than 44 years (3.58/1,000 inhabitants).

Stroke incidence rates were higher than those reported in previous studies in Peru (13/100,000 person/year) and Bolivia (35/100,000 person/year), slightly lower than in Colombia (89/100,000 person/year) [3], and lower than developing countries in Central/Eastern Europe (276.2/100,000 person/year) [31] and rural China (298/100,000 person/year)—a region with one of the highest burdens of stroke [28]. Higher incidence rates have been related to low education and socioeconomic status [28].

If we added the 25 premature stroke deaths to the 157 PWS in our study (182 total PWS), the real prevalence of stroke would have been 8/1000 inhabitants—higher than reported in other prior studies. Limited access to health care, low educational levels, ethnic factors, among other underreported or unidentified factors, may all play a role in Peru’s high prevalence of stroke in rural areas. In terms of numbers, 251,520 inhabitants in Peru reported a stroke event (All Tumbes Region), higher than reported in a previous epidemiological study in Peru (186,000 inhabitants). Other factors specific to different rural areas may also increase stroke risk.

Hypertension (119/157, 75.8%) and sedentary lifestyle (138/157, 87.9%) were associated with stroke in our study; prevalence of hypertension in PWS was similar to that reported in studies in Latin America [3, 6, 7, 32] and Sub-Saharan Africa [33] but lower compared to Nigeria (92.5%) [29]. Hypertension prevalence in our population was 16%, lower compared to Indonesia [34] and European countries (30–45%) [35]. Seventy percent of PWS who had hypertension were poorly compliant with antihypertensive therapy, consistent with information from other Peruvian regions [36]. Sedentary lifestyle is a public health problem worldwide [37]; Every hour of sedentary behavior increases systolic and diastolic blood pressure by 0.06 mmHg and 0.2mmHg, respectively [38]; including a dynamic educational component in regional non-communicable disease programs to promote healthier lifestyles in elementary schools, popular-dining places (comedores Populares), and rural households could be a strategy for encouraging physical activity and help decrease high blood pressure. Improving the patient-health center relationship is essential for empowering the patient on the importance of effective treatment compliance and lifestyle changes to control hypertension and avoid a stroke event.

Twenty-five of 95 deaths reported in our study were due to post-stroke complications. Although our study was not designed to evaluate mortality, our crude mortality rate (1,576/100,000 deaths per year) in people suspected of stroke was extremely high compared to mortality rates from stroke in China (159/100,000 deaths per year) [28] or the US (37.6/100,000 deaths per year) [39]. Low socioeconomic and educational levels and social inequality have been associated with higher mortality rates in other poor regions of Latin America and the Caribbean [2].

This study supported Saposnik and Del Brutto’s hypothesis that a high incidence of stroke is associated with a high risk of mortality during the acute stroke phase [3]. Although a significant proportion of the rural population had access to free health care through the Peruvian government’s SIS (Sistema Integral de Salud) program, many PWS sought initial evaluation through "sobadores" (people who massage the part of the body affected by a stroke) and only later sought traditional care at the hospital outpatient clinic. This particular behavior has been found in other neurological research in this area [40]. This and other cultural idiosyncrasies may delay seeking medical treatment, and future research should examine how improving the patient-health-care provider interaction could reduce such delay. A comprehensive primary-care training campaign to improve education for patients regarding stroke symptoms, prevention, and treatment, as well as optimal cardiovascular health measures, might encourage future stroke patients to seek their first care through hospitals.

Our study’s strengths included the support of 20 GPs from local health centers to recruit and enroll patients, and 8 neurologists to confirm stroke diagnoses. We demonstrated that it is possible to apply the WHO’s suggestions and accomplish stroke detection at the primary-care level, where the main health response is primary-care and prevention [41]. This strategy could be replicable by chronic disease programs inside other ministries of health.

Our study had some limitations. Neuroimaging was limited to CT, which is less sensitive than MRI for detecting stroke. Also, we did not perform EKG to assess for cardiac pathology. It is possible that stroke was under-represented as a cause of death, as “*sudden deaths”* caused by cardiovascular or cerebrovascular disease, are not verified by pathologic evaluation and are often reported as “cardiorespiratory arrest” on death certificates [42]. Epidemiological research with standardized methodology to identify factors associated with high mortality rates in stroke is urgently needed for a more accurate assessment of the burden and characteristics of stroke mortality in Peru.

## Conclusion

Stroke prevalence at sea level in Peru was higher than prior epidemiological studies from South America, but lower than in industrialized countries where the decrease in mortality of stroke has increased survival. High mortality in PWS compared to other rural settings could be due to a lack of care-seeking at primary care levels. Although stroke is the main cause for disability, it will remain a neglected chronic disease, especially in rural settings, until health programs increase services for cardiovascular health, stroke prevention, treatment, disability, and post-stroke rehabilitation.

## Supporting information

S1 Data (XLSX)
